# Development of replication-competent adenovirus based vaccine vectors

**DOI:** 10.1186/1742-4690-9-S2-P310

**Published:** 2012-09-13

**Authors:** P Abbink, LF Maxfield, DH Barouch

**Affiliations:** 1 Beth Israel Deaconess Medical Center, Boston, MA, USA

## Background

Replication-incompetent adenovirus vectors have shown promise as vaccine candidates. We are developing replication-competent adenovirus vectors to increase antigen expression and duration and to facilitate mucosal routes of vaccine delivery. We have developed replication-competent Ad5 (rcAd5) and Ad26 (rcAd26) based vectors, tested their growth in human and simian cell lines, and determined the dynamics of virus shedding after inoculation of rhesus monkeys.

## Methods

Replication-competent Ad5 and Ad26 vectors were produced by adding the E1 region back into the vector. To facilitate rcAd5 growth in rhesus monkey cells, 2 host range mutations were also introduced into the DNA binding protein. The growth of rcAd5 and rcAd26 was tested in human (Per55K, 293, and A549) and simian (CV-1 and Cos7) cell lines. In addition, rcAd5 was administered intranasally to rhesus monkeys, and the kinetics of viral shedding was determined by qPCR on nasal, oral, and rectal swabs, and serum.

## Results

As expected, replication-incompetent Ad5 and Ad26 grew in the E1-complementing cell lines Per55K and 293, but not in A549 or CV-1 cells. In contrast, rcAd5 and rcAd26 grew in all human and simian cell lines tested, although rcAd26 growth was suboptimal in simian cells. After intranasal inoculation of rhesus monkeys, rcAd5 viral sequences could be detected by qPCR in nasal swabs for 5 weeks post-inoculation.

## Conclusion

Replication-competent Ad vectors can be produced efficiently by the re-introduction of E1 into standard replication-incompetent vector backbones. However, the extent of replication in simian cells appears to vary based on Ad serotype. Future studies will compare the immunogenicity of replication-competent vs. replication-incompetent Ad vectors.

